# Real-world analysis of treatment patterns and survival outcome of glioblastoma patients in a German single-center study: Can survival rates of randomized controlled trials be achieved?

**DOI:** 10.1093/noajnl/vdaf009

**Published:** 2025-01-17

**Authors:** Samuel Paus, Johannes Hoffmann, Julia Roeper, Frank Griesinger

**Affiliations:** Department of Hematology and Oncology, Goethe University Frankfurt, University Hospital, Frankfurt am Main, Germany; Department of Hematology and Oncology, Carl von Ossietzky University Oldenburg, University Medicine Oldenburg, University Clinic for Internal Medicine-Oncology, Pius-Hospital, Oldenburg, Germany; Department of Hematology and Oncology, Carl von Ossietzky University Oldenburg, University Medicine Oldenburg, University Clinic for Internal Medicine-Oncology, Pius-Hospital, Oldenburg, Germany; Department of Hematology and Oncology, Carl von Ossietzky University Oldenburg, University Medicine Oldenburg, University Clinic for Internal Medicine-Oncology, Pius-Hospital, Oldenburg, Germany; Department of Hematology and Oncology, Carl von Ossietzky University Oldenburg, University Medicine Oldenburg, University Clinic for Internal Medicine-Oncology, Pius-Hospital, Oldenburg, Germany

**Keywords:** glioblastoma, real-world, German, survival, treatment

## Abstract

**Background:**

The reported survival data for glioblastoma patients vary strongly between different studies. In our study, we therefore examined which data are applicable in a real-world population in a German center and how these real-world data perform in comparison to survival data presented in randomized controlled trials (RCTs).

**Methods:**

Data of all patients treated with newly diagnosed glioblastoma in a single German center between 2010 and 2019 were analyzed and treatment patterns plus survival rates were matched to existing real-world data and results of RCTs.

**Results:**

Two hundred thirty-three patients were analyzed. Median age was 63 years, f/m ratio was 1:162, and 73% of patients underwent surgery, while 27% had biopsy only. The extent of resection had a significant impact on overall survival (OS; *P* <.001), as well as age (*P* <.001), methylguanine methyltransferase methylation status (*P* <.001), and eastern cooperative oncology group performance status (*P* <.001). The median OS of our whole study population was 10.55 months. While a fictitious Stupp study cohort (built by using eligibility criteria of the EORTC-22981-26981 trial) with an OS of 14.3 months nearly achieved the survival results of the presented data from the EORTC-22981-26981 trial, the OS of the patients who did not fulfill the eligibility criteria was only 6.9 months.

**Conclusion:**

Survival of patients with unfavorable prognostic factors is still poor and these patients are not represented in recent RCTs. Outcome data of RCTs can be transferred to real world cohorts, if in- and exclusion criteria are fulfilled, while outcome is significantly inferior in cohorts that do not fulfill these criteria.

**Key Points**

1. Survival outcomes of recent randomized controlled trials (RCTs) can be achieved in a fit subgroup of patients.
2. Still very poor survival outcome for unfit patients.
3. Future RCTs in glioblastoma should also address patients with unfavorable prognostic factors.

**Importance of the study:**

Glioblastoma is one of the most fatal malignancies. Survival rates in literature however vary substantially in parts between the results of some randomized controlled trials (RCTs) and the reported data from real-world populations. For treating physicians, the knowledge about prognosis is yet of particular importance for counseling of their patients and in view of informed decision making. In our study, we describe the survival rates of different subgroups of glioblastoma patients treated in a medium sized German center. We show that the good results in recent RCTs were achieved especially because of strict eligibility criteria, which were fulfilled by only 54.4% of patients in our real-world cohort. Meanwhile, survival rates of patients that do not fulfill eligibility criteria, thus have poorer prognostic factors, and are still very low. This shows the need of more study data especially in the group of patients that do not match eligibility criteria of recent RCTs.

Glioblastoma (GBM) is the most frequent malignant tumor of the central nervous system (CNS) with an incidence of 3-4 cases per 100 000 inhabitants per year^1–3^ and at the same time one of the tumors with the poorest prognosis of all malignancies. Reported overall survival (OS) rates differ substantially between real-world data and recent phase III randomized controlled trials (RCTs), which have led to changes in standard therapy.^4–6^ OS in RCTs ranges from a median survival of 12.1 months in the standard arm of the EORTC-22981-26981 trial^4^ to 48.1 months in the treatment arm of the CeTeg-Studie/NOA-09 trial.^6^ OS in population-based studies lies between 9.2 and 11.7 months^7–12^ and is even shorter for patients above 70 years of age.^7,13^ Well established prognostic markers are age, extend of debulking surgery, performance status, and methylation status of the methylguanine methyltransferase (MGMT) promoter,^10,14–16^ which leads to significant differing OS in these distinct subgroups. The current standard of care for patients <70 years consists of maximal safe debulking surgery with adjuvant radio-chemotherapy and consolidation chemotherapy with six cycles temozolomide.^17^ This so-called Stupp protocol was established after publication of the EORTC-22981-26981 trial.^4^ In 2019 with publication of the CeTeg-Studie/NOA-09 trial, another alkylating agent, CCNU, was added to temozolomide in MGMT-methylated patients.^6^ Treatment recommendations for patients beyond 70 years of age depend on the status of promotor methylation of MGMT. In patients with methylated MGMT either chemotherapy with temozolomide alone or in combination with radiotherapy is recommended, whereas in patients with unmethylated MGMT radiotherapy alone represents standard of care.^17,18^ Despite implementation of treatment stratification based on MGMT methylation and age, there is still a discrepancy between survival rates in recent real-world data and reported outcome of the mentioned RCTs.^11^ As well there is utterly need of better and more precise subgroup stratification. The aim of this study is to identify reasons for the discrepancy between OS in real life and study populations and to give an overview of the epidemiology, treatment sequences, and the outcome of GBM patients in a single German institution.

## Methods

### Population

All patients with histologically diagnosed glioblastoma between January 2010 and December 2019 and who had either debulking operation or biopsy and who received systemic chemotherapy or radiotherapy at the Pius Hospital Oldenburg were recruited in this retrospective cohort study using the tumor documentation system of the hospital. Surgery was performed in external hospitals (in or outside of Oldenburg), diagnosis was confirmed pathologically using the 2007 WHO classification of tumors of the CNS and the 2016 WHO classification, respectively.^19,20^ Cases were discussed for treatment recommendation within the in-house tumor board in cooperation with the Department of Neurosurgery (Protestant Hospital Oldenburg) as well as the Department of Radiotherapy (Pius Hospital Oldenburg). In the mentioned period, 235 patients were identified for the registry with primary or secondary glioblastoma, of whom two patients could not be included since no informed consent was available. Ultimately, the data of 233 patients were analyzed.

### Data Acquisition and Variables

The observation period of this retrospective monocentric study was until the April 24, 2020. All data were collected from the patients’ electronic medical records in the hospital information system at the Pius Hospital Oldenburg. The data used for the retrospective analysis are derived from clinical routine documentation and no additional data were gathered, if data were missing.

Data that were captured for the registry included the following parameters: sex, age at diagnosis, histological diagnosis (glioblastoma multiforme, giant cell glioblastoma, gliosarcoma, glioblastoma with oligodendroglioma component, and gliomatosis cerebri), date of diagnosis (defined as the date of the surgical removal or biopsy), date of death/last contact, performance status of the eastern cooperative oncology group (ECOG) recorded at the first contact at the outpatient service of the department of medical oncology after surgery, extent of surgery obtained from the neurosurgical reports (categorized in biopsy, partial resection which is defined as less than 90% resection of contrast enhancing tumor, gross resection defined as >90% resection of contrast enhancing tumor^21^), type of adjuvant treatment (categorized in chemoradiotherapy [+ chemotherapy consolidation], chemotherapy only, radiotherapy only, none), reasons for discrepancy from standard of care defined as Stupp protocol (age, progressive disease, adverse events, patients will), treatment with Tumor-Treating-Fields-Optune (TTF) (yes or no), number of therapies at recurrence, and molecular markers (IDH mutation tested either immunohistochemical or with Sanger sequencing, MGMT methylation, ATRX loss, EGFR amplification, 1p/19q codeletion).

To compare our real-world data with the results of phase III trials in glioblastoma, we used the three big clinical trials of the past, which had major impact on the standard of care: EORTC-22981-26981, CeTeg-Studie/NOA-09, and EF14 as reference. We defined standard of care as therapy according to the Stupp protocol.

### Ethics

The study was approved by the Medical Ethics Committee of the Carl von Ossietzky University Oldenburg (Reference number: 2019-119). The clinical glioblastoma register implemented in the University Department of internal medicine-oncology at the Pius Hospital Oldenburg as a part of this study was registered in the German register for clinical studies (**DRKS-ID:** DRKS00028497).

### Statistical Analysis

For statistical analyses, we used IBM SPSS statistics version 29.0.0.0. We first conducted a descriptive data analysis of patient characteristics and treatment patterns. They are represented as observed counts and percentages. OS from time of diagnosis (date of primary surgery) to date of death was calculated using the Kaplan–Meier method. We then investigated univariate association of survival in different groups (age, gender, grade of resection, ECOG, MGMT methylation status) by using Cox-Regression. A *P*-value <.05 was considered significant.

Since a new WHO classification of tumors of the CNS has been published in the meantime after the end of the observation period,^22^ we applied the diagnostic criteria of this WHO 2021 classification to our patients for reclassification. Therefore, we excluded all patients with an IDH-mutation or unknown IDH-mutation-status. We also excluded all patients with a histological subtype which is not classified as glioblastoma anymore (glioblastoma with oligodendroglioma component and gliomatosis cerebri). It was not possible to screen for H3.3 G34 mutations subsequently to exclude cases of diffuse hemispheric gliomas, H3.3 G34 mutant, which are considered a new entity since the WHO 2021 classification.^22^ All patients left in this group fulfill the diagnostic criteria of a glioblastoma using the WHO 2021 classification of tumors of the CNS (IDH wildtype and neovascularization or necrosis) besides the H3.3 G34 negativity. The above-mentioned analyses were conducted to this group as well.

In a second step, we excluded all patients from analysis who did not fulfill eligibility criteria for the EORTC-22981-26981 trial, thus worldwide standard of care establishing trial, and again conducted the above-described data analyses for this certain subgroup. Following inclusion and exclusion criteria of the EORTC-22981-26981 trial patients were classified as potentially trial eligible, when the following inclusion criteria were met and had been documented: ≤70 years of age, ECOG ≤ 2, newly diagnosed and histologically confirmed glioblastoma. Because data in our study do not capture any laboratory values, no patients could be classified due to their hematologic, renal, and hepatic function.

We did the same with all MGMT methylated patients for the CeTeg-Studie/NOA-09 trial using its inclusion and exclusion criteria (age ≤ 70 years, ECOG ≤ 1).

## Results

### Patient Characteristics

The population consists of 233 patients of which 89 (38.2%) are women and 144 (62.8%) men, leading to a f/m ratio of 1:1.62. The median age at diagnosis was 63 years with a range from 18 to 85 years. Sixty-six patients (28.3%) were older than 70 years at diagnosis. A good level of physical functioning (ECOG ≤ 2) was reported for 179 patients (76.8%). For 30 (12.9%) patients, no information on the performance status could be found in patient records. The IDH1/2 status was examined in 207 patients (88.7%) of whom 11 (5.3%) showed a mutation. MGMT methylation was tested in 179 patients (76,8 %), in 82 of 179 (45.8%) patients a methylated MGMT promoter was detected, and 67 patients were tested for EGFR overexpression, of whom 42 (62.7%) were tested positive. Loss of ATRX was detected in 8 (7%) of 115 tested patients. Detailed patient characteristics are outlined in Table 1.

**Table 1.** Patient Characteristics

Characteristic	n/n with data (percentage)
Age (median (range))	63 (18–85)
≤70 years	167/233 (71.7%)
**Gender**	
Male	144/233 (62.8%)
Female	89/233 (38.2%)
**ECOG-Performance-Status**	
0	94/203 (46.3)
1	52/203 (25.6)
2	33/203 (16.3)
3	19/203 (9.4)
4	5/203 (2.5)
Data missing	30/233 (12.9)
**Histopathological diagnosis**	
Glioblastoma multiforme	216/228 (94.7%)
Gliosarkoma	4/228 (1.8%)
Giant cell glioblastoma	3/228 (1.3%)
Glioblastoma with oligodendroglioma component	1/228 (0.4%)
Gliomatosis cerebri	4/228 (1.8%)
**Molecular markers**	
IDH1/2 mutation	11/207 (5.3%)
MGMT methylation	82/179 (45.8%)
EGFR amplification	40/67 (59.7%)
Loss of ATRX	8/115 (7%)

### Treatment Patterns

A total of 170/233 (73%) patients underwent primary surgery, while 63 (27%) patients had biopsy only. Gross resection was achieved in 121 cases (71.1% of operated patients). For adjuvant treatment, 160 (68.7%) patients received radio-chemotherapy according to Stupp protocol, five patients (2.1%) according to CeTeg protocol. Patients who started consolidation chemotherapy got a median of five cycles of chemotherapy after finishing the radio-chemotherapy (range 1-19). The biggest subset of 55 out of 165 patients (33.3%) did not receive any cycle of consolidation therapy, indicating that radio-chemotherapy postsurgery had failed before starting consolidation therapy. Only 44/165 (26.7%) patients finished the whole six courses of consolidation TMZ according the Stupp protocol. Reasons for discontinuation of treatment in the radio-chemotherapy group were in 56 cases (34%) progressive disease and in 17 (10.3%) death. Only 12 (7.3%) had to stop treatment because of toxic effects. And 6 (3.6%) patients stopped treatment because of their own decision. Thirty-seven (15.9%) patients received radiotherapy alone and 21 (9%) chemotherapy with TMZ alone. Ten patients (4.3%) did not receive any further treatment after operation. In the age group ≤70 years, treatment was mostly performed independently from MGMT methylation status as recommended in guidelines (*n* = 143, 85.6%). In contrast, patients >70 years were often treated stratified on MGMT methylation status. MGMT methylation status was available in a high proportion of elderly patients 56/66 (84.9%). In this group, 23 (41.1%) had methylated MGMT and 33 (58.9%) had not. From MGMT-methylated elderly patients, 34.8% (8) received radio-chemotherapy and 52.2% (12) chemotherapy only, whereas elderly patients with unmethylated MGMT received mostly radiotherapy only (26 pts, 78.8%). Tumor-Treating-Fields Optune were used in a very small group of 17 (7.3%) patients, accounting for 12.4% of cases since their introduction in 2015. Table 2 outlines these treatment characteristics.

**Table 2.** Patterns of Treatment

Characteristic	n/n with data (percentage)
**Extend of surgery**	
Gross resection	121/232 (52.2%)
Partial resection	48/232 (20.7%)
Biopsy	63/232 (27.2%)
**Adjuvant treatment**	
Radio-chemotherapy	165/233 (70.8%)
Radiotherapy only	37/233 (15.9%)
Chemotherapy only	21/233 (9%)
none	10/233 (4.3%)
**Cycles of adjuvant chemotherapy after RT**	
0	55/162 (34%)
1	13/162 (8%)
2	13/162 (8%)
3	14/162 (8.6%)
4	8/162 (4.9%)
5	4/162 (2.5%)
6	44/162 (27.2%)
≥6	11/162 (6.8%)
**Reasons for discontinuation of concomitant chemotherapy**	
Death	17/165 (10.3%)
Progressive disease in MRI	47/165 (28.5%)
Progressive disease clinically	9/165 (5.5%)
Toxic effects	12/165 (7.3%)
Decision by patient	6/165 (3.6%)
**Further therapy lines (*n* = 223 patients with adjuvant treatment)**	
0	107/223 (48%)
1	52/223 (23.3%)
2	41/223 (18.4%)
3	12/223 (5.4%)
4	9/223 (4.0%)
5	2/223 (0.9%)
**Form of further therapy (*n* = 116 patients with at least 2nd line therapy)**	
Re-operation	47/116 (40.5%)
Reradiation	22/116 (19%)
Rechallenge TMZ	40/116 (34.5%)
Procarbacine/CCNU/(Vincristin)	25/116 (21.6%)
CCNU/TMZ	1/116 (0.9%)
CCNU monotherapy	2/116 (1.7%)
Bevacizumab	52/116 (44.8%)
Nivolumab	10/116 (8.6%)
**Tumor-treating-fields**	
Yes	17/233 (7.3%)

### Survival Outcomes

**Figure 1.**
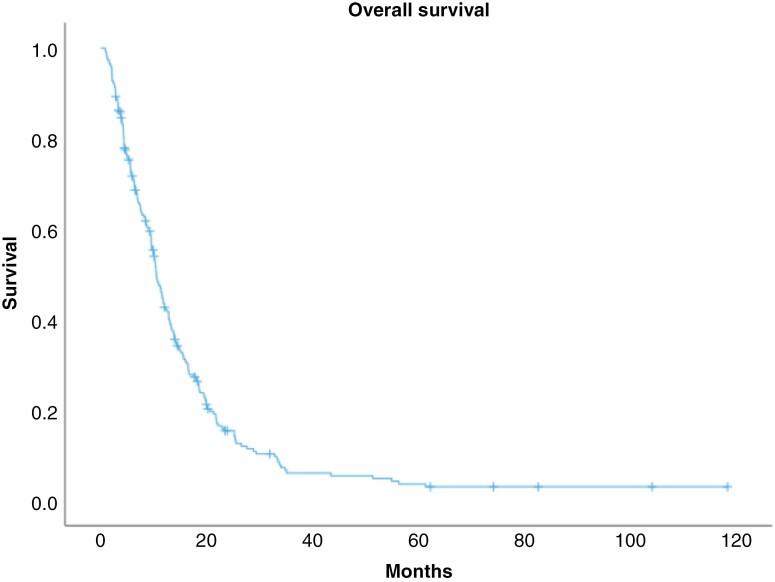
Overall survival.

The median OS of the whole study population was 10.6 months (95% CI [9.3-11.7]) as shown in Figure 1. The 1-, 2-, and 5-year survival rates were 39.5% (92), 11.6% (27), and 2.6% (6) respectively. In the univariate analysis, older age >70 years was associated with shorter survival (hazard ratio 2.21 [95% CI, 1.62-3.01] *P* <.001). Differences in survival between patients regarding varied ECOG scores were significant (*P* <.001) (HR vs ECOG O: 1.94 [95% CI, 1.33-2.83] for ECOG 1, 2.24 [95% CI, 1.49-3.39] for ECOG 2, 4.47 [95% CI, 2.66-7.54] for ECOG 3, and 10.72 [95% CI, 4.15-27.66] for ECOG 4), as well as extent of primary surgery (*P* <.001) (HR vs gross resection: 1.78 [95% CI, 1.24-2.56] for partial resection and 2.73 [95% CI, 1.97-3.79] for biopsy) and MGMT methylation status (*P* <.001) (HR methylated vs unmethylated 0.54 [95% CI, 0.33-0.75]). Table 3 shows survival outcome parameters within these subgroups.

**Table 3.** Survival Characteristics

	Number (%)	Median survival (95% CI) months	*P*-value	Unadjusted hazard ratio (95% CI)
**All patients**	233 (100)	10.5 (9.3-11.7)		
**Gender**				
Female	89 (38.2)	10.3 (7.9-12.8)		
Male	144 (62.8)	10.7 (9.3-12.1)	.882	0.98 (0.74-1.30)
**Age**				
≤70 years	167 (71.7)	12.9 (11.2-14.5)		
>70 years	66 (28.3)	6.9 (4.7-9.2)	<.001	2.21 (1.62-3.01)
**ECOG**				
0	94 (46.3)	15.7 (13.4-18.0)	<.001	
1	52 (25.6)	9.6 (7.9-11.3)	<.001	1.94 (1.33-2.83)
2	33 (16.3)	5.9 (2.9-8.9)	<.001	2.24 (1.49-3.39)
3	19 (9.4)	4.8 (3.9-5.6)	<.001	4.47 (2.66-7.54)
4	5 (2.5)	3.8 (2.7-5.0)	<.001	10.72 (4.15-27.66)
No data	30 (12.9)	7.7 (5.5-9.9)		
**Primary surgery**				
Gross resection	121 (51.9)	13.9 (11.7-16.1)	<.001	
Partial resection	48 (20.6)	10.5 (7.3-13.7)	.002	1.78 (1.24-2.56)
Biopsy	63 (27.0)	5.9 (4.8-7.1)	<.001	2.73 (1.97-3.79)
No data	1 (0.4)	14.8		
**MGMT Status**				
Unmethylated	97 (41.6)	10.1 (9.2-10.9)		
Methylated	82 (35.2)	12.1 (8.2-15.8)	<.001	0.54 (0.38-0.75)
No data	54 (23.2)			
**Study population EORTC-22981-26981**				
Eligible	127 (54.5%)	14.3 (11.6-17.0)		
Ineligible	106 (45.5)	6.9 (5.8-8.1)	<.001	2.71 (2.03-3.63)
**CeTeg-Studie/NOA-09 trial**				
Eligible for control group (Stupp protocol)	39 (16.7)	22.9 (17.6-28.2)		
**WHO 2021 GBM Population**	197	10.4 (9.1-11.8)		

To build an artificial study population out of our real-life cohort, we excluded patients that would not have met eligibility criteria for EORTC-22981-26981 trial, and 66 of 233 patients in our cohort were excluded because of age (>70 years), another 22 patients were excluded due to ECOG score >2. Eventually, 145 patients would have fulfilled the inclusion criteria for EORTC-22981-26981/Stupp protocol, of whom 127 received therapy following Stupp protocol.

**Figure 2.**
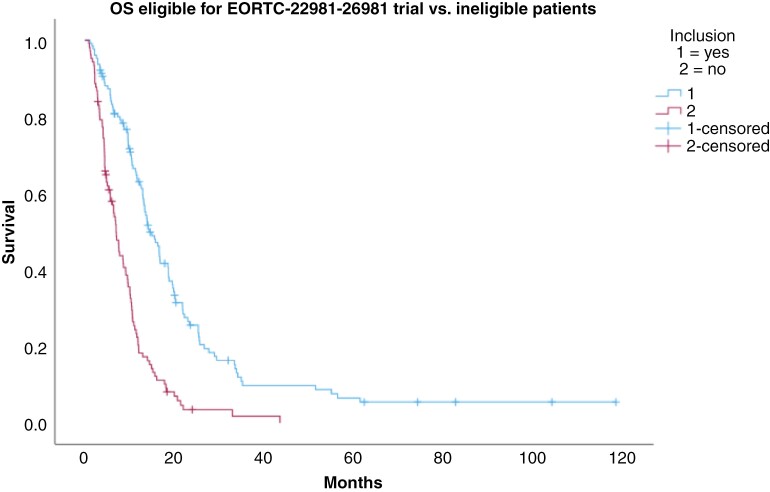
Overall survival in potential study cohort.

The median survival in this subgroup was comparable to the EORTC-22981-26981 trial with 14.3 (11.6-17.0) months. One-, two-, and five-year survival rates were 57.5%, 19.7%, and 4.7%, respectively. The median survival rate of the group of patients who did not fulfill the inclusion criteria was just 6.9 (5.8-8.1) months. The differences in survival are shown in Figure 2.

Applying the eligibility criteria of the CeTeg-Studie/NOA-09 trial (age ≤ 70 years, ECOG ≤ 1) to all MGMT methylated patients in our cohort, a group of 44 patients remained. With only five patients treated following the CeTeg protocol. This is because the NOA-09 trial was just published at the end of enrollment of our study, and 39 patients build an artificial study population comparable to the control group of the CeTeg-Studie/NOA-09 trial (treated following Stupp protocol). The median OS in this group was 22.9 (17.6-28.2) months. One-, two-, and five-year survival rates were 69.2%, 28.2%, and 7.7%, respectively.

The group of patients fulfilling the diagnostic criteria of the WHO 2021 classification of CNS tumors was just 197 patients. Most of exclusions from patients of the primary cohort was because of lacking IDH-mutation status data. The median OS of this group was 10.43 months (95% CI [9.1-11.8]). The one-, two-, and five-year survival rates were 38.6% (76), 7.6% (15), and 1% (2), respectively.

An overview of this survival characteristics is presented in Table 3.

## Discussion

Our study shows that even in a real-world setting of GBM treatment survival outcome of recent phase III clinical trials can be achieved. However, phase III clinical trials are carried out with an assumably highly preselected patient population with eligibility criteria which are not met by a substantial part of patients in our cohort. Examining our whole cohort, survival times are substantially shorter than in recent phase III trials.

The patient cohort of this study in a single center, medium scale German hospital with a big catchment area in north-west Germany differs little in its epidemiological metrics from the data known from big epidemiological studies in GBM. The ratio from women to men is 1:162 compared with 1:157 described in references.^2^ The median age of 63 years at time of diagnosis corresponds to data of epidemiological and other population-based studies (range from 63.3 to 64 years).^1,2,7,23,24^ Aside from that, the distribution of molecular mutation frequency correlates with references in the literature for relevant markers. MGMT was methylated in 46.6%; IDH mutations were prevalent in 5.3% of cases, which is both very similar to values known from literature.^16,23,25^ This indicates that our cohort is representative for the general population of GBM patients classified by WHO 2007 and 2016 classification which was also used in the mentioned clinical trials and other real-world analysis and is not substantially confounded by selection bias.

Nonetheless, we reclassified the patients of our cohort applying the WHO 2021 classification to minimize the risk of overestimating the OS by a high proportion of IDH mutated patients and to make the results comparable to a today GBM population. The median OS of 10.4 months in this group was not shorter than in the primary cohort. While the 1-year survival rate was like the primary population, the 2- and 5-year survival rates were lower. Suggesting that the influence of IDH mutated patients might be high on the proportion of superior survivors in a certain cohort but not on the median OS because of its relatively low rate.

Already well-known prognostic factors such as age, extent of primary surgery, ECOG Performance status, and MGMT methylation status^10,14–16^ showed statistical significant impact on OS in our study as well.

The OS of our whole study population was 10.6 months and thus is in the range of reported data in other recent real-world studies,^7–12,24^ but substantially lower than the OS of 14.6 month achieved in the temozolomide + radiation arm of EORTC-22981-26981 trial or even of 31.4 months in the standard arm (temozolomide + radiation) of the CeTeg-Studie/NOA-09 trial. However, after applying the eligibility criteria of the above-mentioned studies to our cohort, it was apparent that exemplary for the EORTC-22981-26981 trial only 54.5% (127) of patients would have been possible candidates for inclusion. OS of this selective subgroup of our cohort was 14.3 months and therefore not significantly shorter than in the EORTC-22981-26981 trial. This indicates that the differences in OS between phase III trials and real-world data are not based on potentially better medical attendance (eg frequent monitoring and follow-up) but are expression of highly selective eligibility criteria. This could be demonstrated by other studies as well.^8,26–28^

The seemingly low rate of patients in the radio-chemotherapy group completing the whole six circles of consolidation chemotherapy (34%) is not different to other real-world data.^24^ Also, the rate in the artificial Stupp population completing all six cycles was with 37% not very different to the rate in EORTC-22981-26981 trial (47%).^4^ The high rates of discontinuation of treatment due to progress or death is indicating that these high rates are not because the treating physicians do not follow treatment protocols or because of problems with compliance but because of the highly aggressive character of the disease.

In our cohort only, rarely TTF were used (12.4% [*n* = 17] of cases since their introduction in 2015) despite good results of the EF14 trial, showing a survival benefit of the treatment arm with a prolonged survival from 16 to 20.9 months.^5^ Reasons for this relatively small number might lie in the relatively shorter period of only 4 years of observation time since introduction and a complex prescription procedure in the beginning but were not explicitly recorded in our study. This could be a goal for further investigations, since TTF seem to represent a possible improvement in the prognosis of GBM patients not yet utilized.

Selection of further-line treatment in case of refractory/progressive disease was highly heterogenous and different drugs were applied in different sequence. This represents missing guideline recommendations for second-line treatment in GBM.

Our study is limited by the retrospective and single center study design. Only data, which were collected in clinical routine, could be used. Especially examination of ECOG performance status is dependent on examiner and time of examination and was not standardized. It was not possible to review the performance data. And it was also not possible to score patients subsequently by the given information if the ECOG score was not mentioned in the routine clinical data. This led to a relatively high proportion of patients without information about the performance score. As well, data for the results of the extend of primary surgery were just gathered from neurosurgical reports and were not verified independently by postoperative MRI examination.

Also, the data for the different molecular markers are incomplete too. A subsequent molecular analysis was performed in single cases for MGMT methylation but was not possible for most of the cases with missing information. This led to some missing information in the description of the cohort. But the distribution of availability of data of the molecular markers over the years also shows how the diagnostic process in daily clinical routine has changed in the last decade and how fast the WHO 2016 classification and the integrated diagnosis concept was implemented.

## Conclusion

Cohorts in phase III GBM trials consist of a highly preselected group of patients. Study results therefore do not apply to clinical daily routine. Real-world data contribute to provide a completer and more distinct picture and should be considered in treatment decisions and especially individual guidance of patients.

In particular, the prognosis of older patients (OS of 6.9 months in patients >70 years of age in our cohort) is still alarming poor. In case of refractory/progressive disease, a distinct therapy strategy is missing. Future RCTs should urgently address these two groups of patients.

## Data Availability

The data collected in the study will be made available upon reasonable request. The data are stored in an institutional repository and can be provided as an Exel spreadsheet via Dropbox. A secure, password-protected link will be created to grant access to the file.
